# LETTER TO THE EDITOR The Effect of W-plasty on Cheek Rotation Flap

**Published:** 2010-01-04

**Authors:** Masahiro Murakami, Kotoho Oki, Hiko Hyakusoku, Rei Ogawa

**Affiliations:** Department of Plastic, Reconstructive and Aesthetic Surgery, Nippon Medical School Hospital, Tokyo, Japan

Dear Sir,

Cheek rotation flaps^1^^-^3 are used to reconstruct lower eyelids. After rotating the flap, the suture line on the cheek is not aligned with a relaxed skin tension line, and postoperative scars that intersect the margin of the lower eyelid at angles close to 90° may cause downward contractures of the lower eyelid. We describe herein 2 cases in which we avoided this problem by using w-plasty when suturing the cheek rotation flaps to the remaining eyelid and cheek skin in reconstructions, following Meibomian adenocarcinoma resections.

Case 1: The Mustardé method was used to reconstruct the left upper eyelid of a 74-year-old woman after a Meibomian adenocarcinoma was resected. Two weeks after the first operation, the switch flap was detached and w-plasty was performed to close the flap donor site. Two years postoperatively, there was no pronounced scarring and the lower lid had not evinced any contractures due to the suture line (Fig 1).

Case 2: After resecting a Meibomian adenocarcinoma of the right lower eyelid of a 68-year-old woman, the lower eyelid was reconstructed by transferring a cheek rotation flap and suturing it by using w-plasty. One year and 3 months postoperatively, the results were cosmetically favorable, with no downward contracture of the lower lid or conspicuous scarring being observable (Fig 2).

Postsurgery scars are more visible in Asians and Africans than in whites, and w-plasty appears to be a good way to make them less visible. W-plasty has an “accordion effect” in which the tension around a scar is released and dispersed. Randomized controlled trials are necessary to confirm the efficacy of this procedure, but given our positive experiences, we recommend that it be added to the conventional cheek rotation flap method of reconstruction. It is also suggested that w-plasty may be effective for similar flaps that are used to reconstruct the cheek and/or lower eyelids (eg, the cervicofacial flap).^4^,^5^ It will be of interest to determine the utility of w-plasty for these flaps in the future.

## Figures and Tables

**Figure 1:**
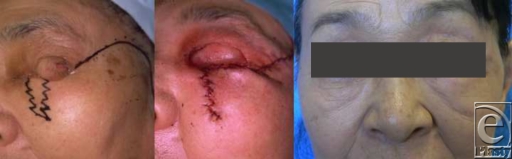
The Mustardé method was used to reconstruct the left upper eyelid. Two weeks after the first operation, the switch flap was detached and w-plasty was performed to close the flap donor site.

**Figure 2:**
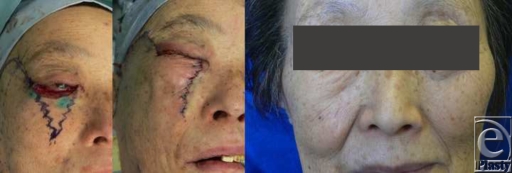
The lower eyelid was reconstructed by transferring a cheek rotation flap and suturing it by w-plasty.
